# *Linnaea borealis* L. var. *borealis*—In Vitro Cultures and Phytochemical Screening as a Dual Strategy for Its Ex Situ Conservation and a Source of Bioactive Compounds of the Rare Species

**DOI:** 10.3390/molecules26226823

**Published:** 2021-11-11

**Authors:** Barbara Thiem, Dariusz Kruszka, Natalia Turowska, Elwira Sliwinska, Viktor Berge, Małgorzata Kikowska

**Affiliations:** 1 Department of Pharmaceutical Botany and Plant Biotechnology, Poznan University of Medical Sciences, 14 Św. Marii Magdaleny, 61-861 Poznań, Poland; bthiem@ump.edu.pl (B.T.); natalia_slow@wp.pl (N.T.); 2 Institute of Plant Genetics, Polish Academy of Sciences, 34 Strzeszyńska St., 60-479 Poznań, Poland; dkru@igr.poznzn.pl; 3 Laboratory of Molecular Biology and Cytometry, Department of Agricultural Biotechnology, Bydgoszcz University of Science and Technology, 7 Prof. Kaliskiego Ave., 85-789 Bydgoszcz, Poland; elwira@pbs.edu.pl; 4 Institute of Clinical Medicine, University of Oslo, Trondheimsveien 235, 0586 Oslo, Norway; viktor.berge@medisin.uio.no

**Keywords:** twinflower, micropropagation, callus, triterpenoid saponins, iridoids, bioactive secondary metabolites

## Abstract

*Linnaea borealis* L. (Twinflower)—a dwarf shrub in the Linnaeeae tribe of Caprifoliaceae family—is distributed across the Northern Hemisphere. By means of this study, a reliable protocol for efficient micropropagation of uniform *L. borealis* L. var. *borealis* plantlets has been provided for the first time; callus culture was also established. Different initial explants, types of cultures, media systems, and plant growth regulators in Murashige and Skoog (MS) media were tested. Agitated shoot cultures in the liquid media turned out to be the best system for the production of sustainable plant biomass. After stabilization of the callus lines, the highest growth index (c.a. 526%) was gained for callus maintained on MS enriched with picloram. TLC and UHPLC-HESI-HRMS analysis confirmed the presence of phenolic acids and flavonoids, and for the first time, the presence of iridoids and triterpenoid saponins in this species. Multiplication of *L. borealis* shoot culture provides renewable raw material, allowing for the assessment of the phytochemical profile, and, in the future, for the quantitative analyses and the studies of the biological activity of extracts, fractions, or isolated compounds. This is the first report on in vitro cultures of traditionally used *L. borealis* rare taxon and its biosynthetic potential.

## 1. Introduction

*Linnaea borealis* L. (Twinflower), a creeping dwarf shrub, was Linnaeus’s favorite plant and became his symbol. This taxon was formerly assigned to the Caprifoliaceae family; however, it was transferred to the family of Linnaeaceae [1,2]. A more recent classification has ascribed twinflower to the Linnaeeae tribe of Caprifoliaceae s.l. [3]. There are the three subspecies recognized within *L. borealis*, which morphologically differ from each other, namely *L. borealis* var. *borealis* in Eurasia, *L. borealis* var. *Americana,* and *L. borealis* var. *longiflora*—both in North America [3,4]. Twinflower has circumboreal distribution, across the Northern Hemisphere, occurring from Scotland and northern Europe through Russia to Siberia, northern Asia to Kamchatka and Japan, northern China and Mongolia, and from Alaska and Canada to Greenland. The main distribution of twinflower in Europe is in the Nordic countries [1,2]. In some areas, the plant holds the ecological value for conservation [5,6,7]. In Poland, as a relic of the Late Glacial period, it reaches the southern extent of its range [8,9]. *L. borealis* is a small creeping evergreen perennial plant with the nature of a dwarf shrub, growing mainly in open pine woodlands [7]. Several factors make natural regeneration of *L. borealis* difficult. Twinflower is clonal self-incompatible and requires cross-pollination to produce viable seeds. Fructification is rare due to long distances between clonal patches. Flowers on different plants are too far apart to be cross-pollinated by insects. As a consequence, twinflower is rarely propagated generatively—seeds are often not produced or germination does not occur. Moreover, the sexual method does not guarantee obtaining uniform, true-to-type plants. *L. borealis* plants often intensively spread in forest stands by above-ground runners known as stolons, which consist of two types of stems: flowering and assimilation shoots. Stolons also produce branches, forming large clonal patches consisting of groups of plants, which are genetically identical. Greenhouse propagation via stem cuttings has been used for horticultural production; however, propagation efforts are often unsatisfactory due to the frequent failure of forming roots or root rot. Attractive flowers make twinflower a suitable commercially-used ground cover plant. Due to the presence of valuable secondary metabolites, this taxon can be interesting for the pharmaceutical and cosmetic industries. Micropropagation may be a tool adopted to help propagation of the valuable species [3,7,9,10].

To our knowledge, the chemical profile of the European subspecies of *Linnaea borealis* var. *borealis* has not been studied so far, while the chemical composition of the American subspecies of *L. borealis* was briefly described in a doctoral dissertation at the University of Colombia [11]. Until recently, little has been known about the chemical constituents. The following flavonoids were detected in the ethanol extracts from leaves: glycosides of quercetin (quercetin 3-*O*-rhamnoglucoside, quercetin 3-*O*-glucoside) and kaempferol (kaempferol 3-*O*-glucoside), as well as apigenin and luteolin derivatives (apigenin 7-*O*-glucoside, apigenin 7-*O*-rhamnoglucoside and luteolin 7-*O*-glucoside). Among phenolic acids, Glennie [11] identified several compounds, namely *p*-coumaric acid (4-hydroxycinnamic), *p*-hydroxybenzoic acid, caffeic acid (3,4-dihydroxycinnamic), ferulic acid (3-methoxy-4-hydroxycinnamic), protocatechuic acid (3,4-dihydrooxybenzoic), vanillic acid (3-methoxy-4-hydroxybenzoic), phloretic acid (4-hydroxydihydrocinnamic), and four chlorogenic acid isomers (3-caffeoylquinic, 4-caffeoylquinic, 3,4-caffeoylquinic, 4,5-caffeoylquinic). The floral scent composition of *L. borealis* comprises about 26 chemical compounds, identified as monoterpenes, benzoids and phenylpropanoids, aliphatics, sesquiterpenes, and irregular terpenes [12]. Furthermore, the floral scent has been described as almond-like or anise-like and consists of four benzoid compounds, i.e., 1,4-dimethoxybenzene, anisaldehyde, 2-phenylethanol, and benzaldehyde, and also one nitrogen-containing compound—nicotinaldehyde [5,11].

In Norwegian traditional medicine, *L. borealis* has a long tradition as a cure for shingles (*Herpes zoster*). In the past, this species was also used in the European countries to treat skin diseases and other kinds of rash, eczema, measles, hives, ringworm, scabies, water blisters, rheumatism, and finger infections. Twinflower was also applied as a medicinal and food plant by indigenous American people [13]. In fact, the studies have already been compiled in the ethnobotanical elaboration [2,14].

The availability of plant material may be limited due to the specificity of climatic and habitat requirements, progressive degradation of the natural environment, as well as slow plant growth and sometimes several-year-long formation of organs constituting valuable raw material. The availability of the quantity of plants growing in the wild is also significantly limited due to their strict or partial species protection. Increasing pollution and unfavorable changes in the natural environment result in the depletion of plant resources, and collection of raw materials from such areas becomes problematic. An alternative solution to these limitations may be the possibility of chemical research and production of desired metabolites in plant biomass obtained with the use of the biotechnological methods. Plant in vitro cultures can provide the sufficient quantity of high quality uniform biomass under controlled conditions and affect secondary metabolites production in the medicinal species. Furthermore, in vitro cultures allow to avoid problems related to the collection, transport, and storage of plant material, which is available all year round. Moreover, micropropagation provides renewable raw plant material, allowing for the assessment of the phytochemical profile, quantitative analyses, and studying the biological activity of extracts, fractions, or isolated compounds [15,16,17].

The aim of the current work was to develop an efficient protocol of *Linnaea borealis* L. var. *borealis* micropropagation by the development of axillary buds and multi-shoot culture, as well as induction of callus from various plant explants and optimization of growth of callus on the different media. The study also aimed at the multiplication of in vitro plants for the preliminary phytochemical assessment for comparison with intact plants. Additionally, the study of propagation was undertaken not only for twinflower conservation, but also for the further ornamental and pharmacological application. So far, there has been no report available on *L. borealis* var. *borealis* in vitro cultures and detailed phytochemical screening.

## 2. Results

In present study, the efficient micropropagation protocol of *Linnaea borealis* var. *borealis* was established using the method of stimulation of new buds from pre-existing meristems (nodal segments or shoot tips with apical meristems). The influence of the type of the plant explant, hormonal supplementation in the medium, and culture system (solid or liquid) on shoot multiplication were estimated (Table 1, Table 2, Table 3 and Table 4). In the next step, the effect of the auxin type/combined auxins on *L. borealis* shoot rooting was evaluated (Table 5). As the artificial conditions of in vitro systems may trigger the so-called somaclonal variation in the propagated plantlets, therefore it was necessary to monitor the genetic fidelity of the clones (Table 6).

### 2.1. Shoot Multiplication

The presence of BAP (first-generation synthetic cytokinin) in the multiplication medium had a particularly positive impact on the development of new shoots from explants in the form of multi-shoots. In turn, the addition of kinetin (also artificial cytokinin) to the multiplication medium was important for the development of new shoots from the nodal fragments of stems. Double shoots, shoot segments with one node, and single shoots with the apical meristem were the best explants for multiplication of shoot biomass. The media with the high cytokinin content were crucial for the development of new shoots. The highest number of new shoots (7.63 ± 0.73) was obtained from double shoots on the solid medium supplemented with BAP (2.0 mg/L), IAA (0.1 mg/L) and GA_3_ (1.0 mg/L), as well as from stem fragments with nodes cultured on the solid MS medium supplemented with BAP (1.5 mg/L), IAA (0.1 mg/L) and GA_3_ (1.0 mg/L), and MS with Kin (2.0 mg/L), IAA (0.1 mg/L), and GA_3_ (1.0 mg/L)—6.13 ± 0.64. The media enriched with GA_3_ were used for elongation of shoots (Table 1).

**Figure 1:**
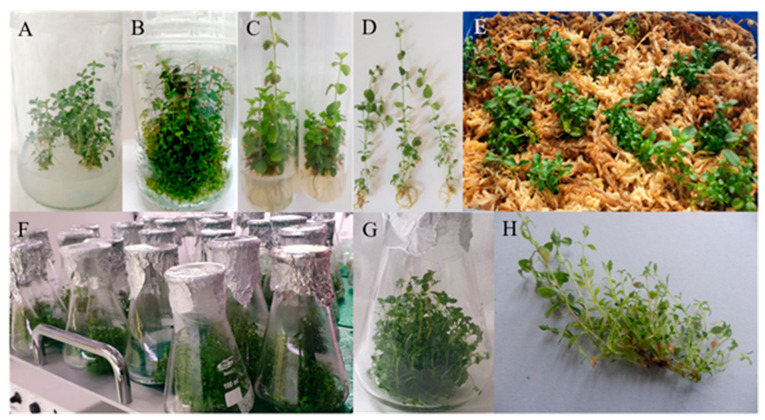
Micropropagation of *Linnaea borealis* var. *borealis* (**A**) shoots on the solidified medium; (**B**) multiplied shoots on the solidified medium; (**C**) rooted shoots; (**D**) rooted shoots before acclimatization; (**E**) micropropagated plantlets hardened in a glasshouse; (**F**) micro-shoots in the liquid media on a rotary shaker; (**G**) multiplied shoots in the liquid medium obtained from double shoots; (**H**) multiplied shoot obtained from one shoot explant from the liquid media.

Micro-shoots were morphologically proper, not vitreous, and did not form callus at the base; leaves exhibited a vivid green color (Figure 1).

In the subsequent experiment, explants with a different number of shoots (single, or a cluster of double or triple shoots) were placed on the selected solid medium—BAP (1.0 mg/L), IAA (0.1 mg/L) and GA_3_ (1.0 mg/L). A double shoot indicated to be the best explant for multiplication. The highest number of new micro-shoots per explant was 10.89 ± 0.55 and 5.37 ± 0.26 per shoot (Figure 1, Table 2).

Admittedly, double and triple shoots grown in the liquid medium proved to be the best material for multiplication of new shoots (their number increased up to about 40); double and single shoots increased the most in length (by about 150%). Therefore, the results of the study have suggested that double shoots are the best explants for liquid culture to obtain a large number of shoots that grow significantly in length, as they generally contribute to the greatest increase in plant biomass. The authors believe that a large number of new shoots is associated with rinsing the whole explant in the liquid medium, which may affect initiation and the development of buds forming new lateral shoots. Well-expanded leaves of cultured shoots had correct morphology and did not show any changes (Figure 1, Table 3).

Summarizing the results of a series of experiments, the introduction of *L. borealis* L. var. *borealis* into in vitro cultures enabled rapid clonal multiplication. Homogeneous plant material was obtained by stimulating the development of axillary buds on the MS medium variant wit BAP 1.0 mg/L + IAA 0.1 mg/L + GA_3_ 1.0 mg/L. The most efficient multiplied shoot biomass was obtained in agitated liquid culture (Table 4).

### 2.2. Shoot Rooting

It was extremely difficult to produce rootlets in the experiment, as twinflower is a dwarf shrub. The first roots were observed after two weeks of culture; after another four weeks, roots were well developed. In vitro-multiplied shoots formed vigorous roots of a white color with frequency of about 30–100%, depending on the applied medium. The highest percentage of root induction of in vitro multiplied shoots was obtained for shoots grown on the solid MS medium with higher concentrations of IBA alone or in combination with IAA. Additionally, these media were advantageous for the number of emerging roots, most of which, about 19 roots per shoot, appeared on the following media: MS + IBA 4.0 mg/L and MS + IBA 2.0 mg/L + IAA 1.0 mg/L. The least preferred media for rooting of shoots were those that lacked IBA and those in which IAA was present at a low concentration. Micro-shoots underwent direct root formation, without a callus phase on all the tested media (Figure 1, Table 5).

### 2.3. Genome Size Estimation

**Figure 2:**
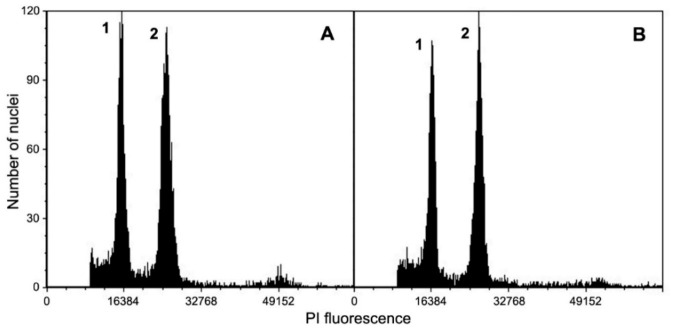
Histograms of the nuclear DNA content obtained after the flow cytometric analysis of the PI-stained nuclei isolated simultaneously from leaves of *Linnaea borealis* var. *borealis* (peak 1), seedling (**A**) and a micropropagated plant (**B**), and *Petunia hybrida* (the internal standard; peak 2).

Micropropagation of *L. borealis* var. *borealis* was performed through meristematic tissues; however, the genome size estimation was necessary to ensure that plant material is homogeneous. The 2C DNA content of leaves in control seedlings was 1.778 pg and it was similar in multiplied shoots (Table 6; Figure 2). No statistical differences were detected.

### 2.4. Callus Induction and Maintenance

The influence of the type of the plant explant and hormonal supplementation in the solid medium on callus induction and proliferation were estimated (Table 7).

**Figure 3:**
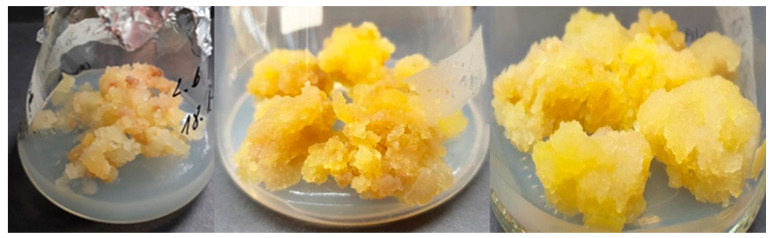
Leaf-derived callus of *Linnaea borealis* var. *borealis* cultured on MS medium enriched with picloram 2.0 mg/L.

Leaves, internode stems, and roots of *L. borealis* var. *borealis* previously multiplied in vitro were used as explants in the experiment. Callus was observed in all the three types of explants, usually at the point of the organ cut. Formation of callus on leaves lasted from four to eight weeks, while on fragments of stems and roots, it took six to 12 weeks. Callus forming on plant explants was light yellow and milky. It was passaged every five weeks until stabilized (VII, VIII, IX passages). As the observations showed, the mean callus induction from leaves was 39.37%, from stems—37%, and from roots—45.5%. After stabilization of the callus lines, the highest growth index gain was characteristic of callus culture maintained on MS medium enriched with picloram 2.0 mg/L (Figure 3, Table 7).

### 2.5. UPLC-HESI-HRMS Phytochemical Screening

**Figure 4:**
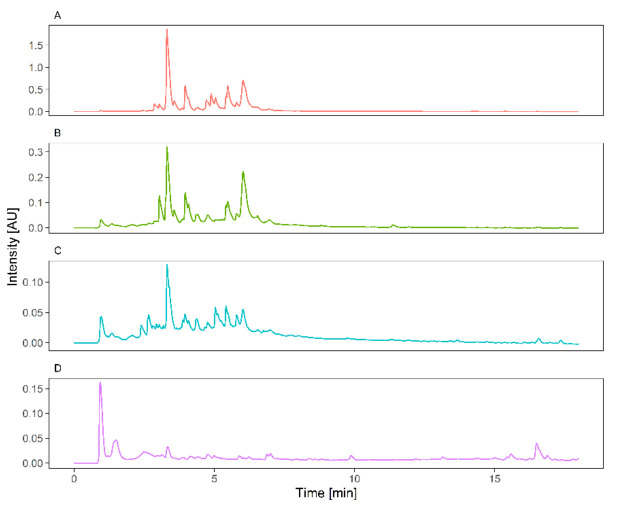
The UPLC-PDA (254 nm) chromatograms of *Linnaea borealis* var. *borealis* extracts of leafy shoots from natural sites (purple, **D**); shoot cultures (red, **A**); roots from micropropagated plantlets (green, **B**); biomass from callus cultures (blue, **C**).

UPLC-MS/MS analysis demonstrated in the studied material, namely leafy shoots of intact plants, shoots and roots of micropropagated plantlets, and callus biomass, the diversity of chemical compounds with known pharmacological activity (Figure 4, Figures S1–S4, Table 8) [18,19,20,21].

The results indicated the presence of the four major groups of secondary metabolites in the studied plant material of *L. borealis* L. var. *borealis*. The presence of iridoid and triterpene compounds, apart from phenolic acids and flavonoids, has been detected for the first time. UPLC-MS/MS analysis in the negative and positive ion mode indicated the presence of 30 phenolic (including benzoates and hydroxycinnamic acid derivatives) and 19 flavonoid compounds. Thirteen iridoid compounds have been detected for the first time in this species. Two organic acids (quinic and pantothenic acid) were annotated in the profile. Additionally, 14 triterpenoid saponins were confirmed with the use of mass spectrometry (Table 8).

In this study, the profile of hydroxycinnamic acid derivatives was mainly presented by esters of quinic acid with caffeic, coumaric, and ferulic acids. Four caffeoylquinic acids were recognized as trans-3-caffeoylquinic, trans-5-caffeoylquinic, trans-4-caffeoylquinic, and cis-5-caffeoylquinic. As reported by Ramabulana et al. [22], isomers of caffeoylquinic were recognized by the characteristic fragmentation pattern following at *m*/*z* value 191.0551 (C_7_H_11_O_6_^−^, 1.5 ppm), 179.0339 (C_9_H_7_O_4_^−^, 0.87 ppm), and 161.0233 (C_9_H_5_O_3_^−^, 0.5 ppm) and retention time scoring. Deprotonated molecule at *m*/*z* 337.0929 were annotated as 3-coumaroylquinic acid (rt = 4.99 min) and 5-coumaroylquinic acid (rt = 5.49 min). Ions at *m*/*z* 367.1038 were observed at rt 5.36 and 5.77 min and putatively marked as 3- and 5-feruloylquinic acids, respectively. The fragmentation pattern showed the loss of ferulate moiety—176.0439 (C_10_H_8_O_3_, 5 ppm). Deprotonated molecule at *m*/*z* 515.1191 (C_25_H_23_O_12_^−^, 1.5 ppm) and 517.1334 (C_25_H_25_O_12+_, 2.29 ppm) were observed for rt of 6.33, 6.46, 6.58, 6.84, 7.57, and 9.05 min, which suggested the existence of a few regio-isomers of dicaffeoylquinic acid. The fragmentation spectra of these precursor ions showed the following fragments in negative ion mode: 353.0876 (C_16_H_7_O_9_^−^, 0.94 ppm), 191.0551 (C_7_H_11_O_6_^−^, −2.53 ppm), 179.0339 (C_9_H_7_O_4_^−^, 3.15 ppm), and 173.0444 (C_7_H_9_O_5_^−^, −3.34 ppm), with varied abundance for specific isoforms. Four forms of caffeoylcoumaroylquinic acid were tentatively identified as 3-caffeoyl-5-coumaroylquinic acid (rt = 7.13 min), 3-caffeoyl-4-coumaroylquinic acid (rt = 7.31 min), 5-caffeoyl-4-coumaroylquinic acid (rt = 7.48 min), and 4-caffeoyl-5-coumaroylquinic acid (rt = 8.18 min). Moreover, conjugates of ferulic acid with caffeoylquinic acid were detected in the extracts and identified as 3-caffeoyl-5-feruloylquinic acid (rt = 7.42 min) and 4-caffeoyl-5-feruloylquinic (7.72 min). An ion at *m*/*z* 341.0875 corresponded with C_16_H_17_O_9_^−^ (2.8 ppm) formula and gave the fragmentation patterns of 179.0339 and 135.0437, characteristic of caffeic acid hexoside. The free form of caffeic acid was found at 8.42 min. One lignan and coumarin were putatively annotated as S(8-8)S hexoside and esculin, in accordance with the exact mass and the fragmentation pattern. Furthermore, derivatives of benzoic acid were found and noted as hydroxybenzoic acid hexoside (two forms at *m*/*z* 299.0771, at rt: 3.43 min and 3.81 min), dihydroxybenzoic acid hexoside (*m*/*z* 315.0723, at rt: 3.67 min), and dimethoxyhydroxybenzoic acid hexoside (*m*/*z* 359.0981, at rt: 3.58 min).

The second group of phenolic compounds were flavonoids. This group was represented by glycoconjugates of quercetin (301.0352, C_15_H_9_O_7_^−^, 1.5 ppm), kaempferide (299.0558, C_16_H_11_O_6_^−^, 0.86 ppm), kaempferol (285.04050, C_15_H_9_O_6_^−^, 3.98 ppm), luteolin (285.0404, C_15_H_9_O_6_^−^, 3.68 ppm), and apigenin (269.0455, C_15_H_9_O_5_^−^, 5 ppm). Four of these compounds, i.e., apigenin, kaempferide, kaempferol, and luteolin, were identified as free aglycones at retention time of 9.10, 8.13, 9.42, and 9.26 min. Quercetin derivatives included quercetin-3-*O*-rutinoside (609.1459, C_27_H_29_O_16_^−^, 0.63 ppm, rt = 5.76 min) and two quercetin hexosides, namely quercetin-3-*O*-glucoside (463.0887, C_21_H_19_O_12_^−^, 2.29 ppm, rt = 6.02) and quercetin-3-*O*-galactoside (463.0887, C_21_H_19_O_12_^−^, 2.29 ppm, rt = 6.25). In compliance with the fragmentation pattern, kaempferol-3-*O*-rutinoside and kaempferol-3-*O*-glucoside were putatively noted in the extracts. The conjugates of flavones were identified as luteolin-7,5-*O*-diglucoside, luteoloin-*O*-rhamnoglucoside (two forms), luteolin-7-*O*-glucoside, apigenin-7-*O*-apioglucoside, apigenin-7-*O*-rhamnoglucoside, and apigenin-7-*O*-glucoside. Neutral losses corresponded with hexose (−162.0527, C_6_H_10_O_5_) and rhamnohexoside (−308.1109, C_12_H_20_O_9_) moiety. The dimeric forms of procyanidin at *m*/*z* 577.1349 (C_30_H_25_O_12_^−^, 1.5 ppm) were observed at given rt: 3.32, 3.49, and 4.71 min. The fragmentation spectra showed characteristic fragmentation pattern: 407.0768, 289.0717, 245.0813, 125.0229. Similarly, the structure 5 was annotated as catechin or epicatechin (isomer forms) that fragmented to *m*/*z* 245.0813, 125.0229.

Iridoid and secoiridoid groups were represented by 13 compounds. Deprotonated molecules at *m*/*z* 375.1294 at 4.11 and 4.27 min, corresponded with a molecular formula C_16_H_23_O_10_^−^ (0.72 ppm). The fragmentation spectra of these precursor ions showed masses of 213.0761 and 151.0752 that corresponded with the loss of hexose moiety and future fragmentation of aglycone. These compounds were annotated as loganic acid and epi-loganic acid. The derivate of loganic acid, deoxyloganic acid, was found at 5.97 min. The ions *m*/*z* 373.11377 (C_16_H_21_O_10_^−^, 2.27 ppm) were found at 4.09 and 4.62 min and annotated as swertiamarin and geniposidic acid. In accordance with literature data, the obtained fragmentation spectra for compounds 27, 29, 30, and 32 were compared with proposed fragmentation models and were noted as loganin (*m*/*z* 391.1591, C_17_H_27_O_10_+, 3.39 ppm), secoxyloganin (*m*/*z* 427.1203, C_17_H_24_O_11_Na^+^, 3.19 ppm), swerioside (*m*/*z* 381.1151, C_16_H_22_O_9_Na^+^, 2.77 ppm), and secologanin (*m*/*z* 389.1437, C_17_H_25_O_10_^+^, 2.76 ppm) [23]. The fragmentation spectra of these precursor ions show the loss of hexose (−162.0527, C_6_H_10_O_5_) and fragmentation of aglycone. The compound 29 was putatively described as grandifloroside in comparison with its MS/MS spectra and exact mass. Moreover, two [M − H]^−^ ions *m*/*z* 585.2195 at 7.54 and *m*/*z* 583.2036 at 7.98 min were marked as unknown terpene glycosides. Several triterpene saponins were found in the plant extracts. These compounds were recorded as macranthoidin A, akebiasaponin D, loniceroside C, bourneioside B, cauloside C, alpha-hederin, and cauloside A. Furthermore, three free aglycones were recognized with the use of the exact mass. These compounds could be annotated as hederagenin (15.14 min), gypsogenin (15.66 min), and oleanonic acid (17.32 min). Four compounds were marked as unknown saponins.

## 3. Discussion

To the best of the authors’ knowledge, this study has shown *L. borealis* introduction into in vitro cultures and the development of the micropropagation protocol, as well as establishment of callus induction and maintenance for the first time.

Micropropagated plants and biomass from the other types of in vitro cultures can be an alternative source of secondary metabolites with the biological activity, which is particularly important for rare or unavailable in nature taxa, for example, due to the species protection status. Biotechnological production of biomass of such species facilitates the phytochemical and biological research without destroying their natural environments. Plant in vitro cultures can provide the sufficient quantity of high quality uniform biomass under controlled conditions [15,16,24].

Qi et al. [25], in their work on micropropagation of the species also belonging to Caprifoliaceae family, i.e., *Lonicera edulis*, indicated that in vitro propagation of multi-shoots promotes rapid multiplication of plant material and can be used in mass reproduction. The authors obtained the most efficient shoot propagation on a similar MS medium with the addition of BAP (1.0 mg/L) and IBA (0.2 mg/L) [25]. Similarly, in in vitro shoot cultures of *Lonicera caerulea* var. *kamtschatica* Pojark., the presence of BAP also enhanced production of new micro-shoots; however, its high concentration (2.0 mg/L) resulted in callus formation at the base of shoots, which is an undesirable feature for the homogeneity of the plant material [26]. This cytokinin at a lower concentration (0.1 mg/L) influenced significant growth of *Lonicera periclymenum* L. [27]. It is widely known that BAP positively affects the development of axillary buds, especially when combined with auxin of much lower concentration [28,29,30]. In the present study, multiplied shoots of *L. borealis* were not vitreous, regardless of the concentration of cytokinin, which is a desirable feature for the quality of plant material. Nonetheless, in the work of Dziedzic [26], it was observed that the media supplemented with a higher concentration of BAP (2.0 mg/L) brought a high percentage (even 36%) of vitrified shoots in *L. caerulea* var. *kamtschatica*. In a protocol of other micropropagated shrubs from Caprifoliaceae, namely *Kolkwitzia* and *Weigela*, the employment of BAP with a low concentration of auxin was preferred for the stimulation of the development of new buds [31,32].

Shoots cultured in the liquid media are a good source of biomass for the phytochemical and biological studies. Shoot biomass of *L. borealis* grew because not only horizontal fragments of stems (which came in contact with the solid medium in the solidified culture systems), but also the nodal segments in vertical stems, poured with the liquid medium, were exposed to the medium nutrients and phytohormones. Agitation clearly promoted shoot growth in liquid cultures of some other species, for example, *Eryngium alpinum* [17], *Lychnis flos-cuculi* [33], *Scutellaria alpina* [34], and *Schisandra chinensis* (Turcz.) Baill. [35]. Interestingly, shoots of the studied *L. borealis* had correct morphology, while agitated shoots from the liquid media of many plant species cultured in vitro were characterized by abnormality and hyperhydricity, for example, *E. alpinum* [17] or *S. alpina* [34].

Micro-shoots of woody plants are usually difficult to grow roots [36]. The use of MS medium supplemented with IAA (5.0 mg/L) and IBA (2.0 mg/L) was dictated by the report on shoot rooting of the woody species, *L. caerulea* var. *kamtschatica*, grown in in vitro cultures. The same types and concentrations of two auxins were used in rooting of *L. borealis* shoots; however, MS medium was used in this study. In the research of Dziedzic [26], Woody Plant Medium (WPM) was applied. Moreover, satisfactory rooting was also obtained for other honeysuckle plants [36,37].

Explants from shoot cultures and plants of *L. borealis* from in vitro culture were used for callus induction; these were leaves, apical fragments of rootlets, and internodal fragments of stems. The highest percentages of responses were obtained from roots, while the most intense callus development was observed on leaves. The differentiation in callus induction from distinct plant explants was affected by the type and age of an explant [38].

The purpose of the preliminary phytochemical analysis was to investigate whether in vitro plant cultures of *L. borealis* var. *borealis* are capable of biosynthesis of secondary metabolites. Multiplied shoots obtained from in vitro cultures produced secondary metabolites analogous to those produced by plants growing in the wild. The analysis of the biomass extracts of callus cultures revealed that callus produced phenolic acids, but was not able to biosynthesize flavonoids. The reason for this inability may be the type of a nutrient solution and a concentration of plant growth regulators used in the medium. The results of other authors indicated that callus cells of various plant species usually do not produce flavonoids, but are able to synthesize phenolic acids [28,39].

The analysis with the use of HPLC/MS has allowed to observe the presence of iridoid compounds and triterpenoid saponins in the European species *L. borealis* var. *borealis* for the first time. The *Lonicera* genus as well as *L. borealis* species belong to the family of Caprifoliaceae s.l., hence, there was a high probability of finding iridoids in biomass of the species studied [40]. Compounds annotated as loganin, secoxyloganin, secologanin, secologanin, loganic acid, and morroniside were present in *Lonicera* species—*L. morrowii*, *L* × *bella*, and *L. tatarica* [40]. The phytochemistry of *Lonicera* was previously investigated due to the importance of various species in traditional pharmacopeias, and the genus contains a class of secondary compounds, iridoid and secoiridoid glycosides, with the established economic importance [40]. According to Jensen et al. [41], secologanin and morroniside—iridoids of VI group (simple secoiridoids), sweroside—iridoid of VII group and loganin—iridoid of X group are characteristic of the family of Caprifoliaceae.

Several triterpene saponins were found in the studied extracts of *L. borealis*. Few triterpenoid compounds were formerly reported in closely related *Lonicera* species. These compounds were annotated as hederin-type triterpenoid saponins macranthoidin A found in *L. confuse* [42], akebiasaponin D and cauloside C determined in *L. macranthoides* [43], as well as loniceroside C and cauloside A revealed in *L. japonica* [44,45]. Moreover, lupine-type triterpenoid saponin—bourneioside B present in the studied *L. borealis*—was previously detected in *L. bournei* [46].

The presence of a larger number of phenolic acids and flavonoids similarly to the study of Glennie [11] was detected. In present study, the profile of hydroxycinnamic acid derivatives was mainly presented by esters of quinic acid with caffeic, coumaric, and ferulic acids. The second group of phenolic compounds were flavonoids. This group was represented by glycoconjugates of quercetin, kaempferide, kaempferol, luteolin, and apigenin, which were partially identified by Glennie in American varieties of *L. borealis* [11]. Research manuscripts reporting large datasets that are deposited in a publicly available database should specify where the data have been deposited and provide the relevant accession numbers. If the accession numbers have not yet been obtained at the time of submission, please state that they will be provided during review. They must be provided prior to publication.

## 4. Materials and Methods

### 4.1. Plant Material, In Vitro Cultures Initiation, and Growth Chamber Parameters

Healthy and vigorous shoot pieces obtained from adult plants of *L. borealis* L. var. *borealis* were collected from the mixed coniferous forest in Wisełka, the Wolin National Park, Poland, in July 2017. The plant specimen was deposited in Herbarium of the Department of Pharmaceutical Botany and Plant Biotechnology of Poznan University of Medical Sciences. About 5–7-cm long fragments of shoots with nodes were thoroughly rinsed with distilled water and then immersed in 70% (*v*/*v*) ethanol for 30 s. Then, plant fragments were washed with a 30% solution of a commercial disinfectant (Domestos), containing 4.28% of calcium hypochlorite with the addition of Tween 20. Sterilization was carried out for 15 min. Explants were thoroughly rinsed with sterile distilled water in a laminar flow cabinet, dried and divided into smaller apical and nodal fragments. After the final wash, individual explants were transferred to 250-cm^3^ Erlenmeyer flasks containing 50 cm^3^ of the solidified basal medium consisting of MS medium [47] supplemented with various plant growth regulators (PGRs), 0.76% agar and pH set to 5.8 prior to autoclaving at 121 °C, 105 kPa for 20 min. The culture vessels were placed in a growth chamber (21 °C ± 2 °C; with a 16 h light/8 h dark photoperiod; 55 µmol/m^2^ s light).

### 4.2. Shoot Multiplication on Solid Media

In the first experiment, the authors of the study tried to find out whether node segments or shoot tips with apical meristems (both of 2–2.5 cm with 3–4 nodes) were more favorable explants for shoot multiplication in relation to double shoots (a cluster consisting of 2 short shoots). To determine beneficial conditions for shoot multiplication, the media were supplemented with BAP (0.5–2.0 mg/L) or kinetin (Kin 1.0 mg/L, 2.0 mg/L), IAA (0.1 mg/L), and gibberellic acid (GA_3_ 1.0 mg/L). Multiplication of shoots was replicated three times for each hormonal treatment, using at least 10–20 explants.

The second experiment was dedicated to verify how many new shoots may be obtained as a result of shoot multiplication within eight weeks. For this purpose, single shoots as well as multi-shoots (with two or three shoots), obtained from the initially multiplied shoots, were transferred to the solid MS medium with the selected concentrations of PGRs, that is BAP (1.0 mg/L), IAA (0.1 mg/L) and GA_3_ (1.0 mg/L). Due to a particularly small size of a plant, explants used for the experiments were not only single shoots, but also clusters of two or three micro-shoots. Multiplication of shoots was replicated three times using at least 10–20 explants.

After a few subcultures, the multiplication rates were recorded by determination of the number of new micro-shoots that proliferated from the initial explant.

### 4.3. Shoot Multiplication in Liquid Media (Agitated Cultures)

Single shoots as well as multi-shoots (with two or three stems) about 2–3 cm long with 5–6 nodes, obtained from developed shoot cultures on the solid media, were transferred to the liquid MS medium with the selected concentrations of PGRs, namely BAP (1.0 mg/L), IAA (0.1 mg/L), and GA_3_ (1.0 mg/L); 100-cm^3^ Erlenmeyer flasks with 10 cm^3^ of medium were used for shoot biomass production (the ratio of glass volume to medium volume was 10:1). Cultures were maintained on a rotary shaker (110 rpm). After eight weeks of culture, the number of new shoots per explant and the shoots length index (LI) were measured. At least 10 explants were used for multiplication of shoots. The initial (L0) and the final (LX) lengths of cultured shoots were measured. The length increase index [LI] was calculated according to the following formula: LI = [(LX − L0)/L0] × 100%.

### 4.4. Shoot Rooting

An attempt at rooting of clusters of several shoots was made. Multi-shoots (3–4 shoots) 3 to 5 cm in length were used. Explants were kept in 70-cm^3^ glass tubes containing 15 cm^3^ of the solidified medium (¼ MS—salts reduced to 25%, ½ MS—salts reduced to 50%, MS—full strength of mineral salts) without auxins or supplemented with IAA (0.5–4.0 mg/L) or indole-3-butyric acid (IBA 0.5–5.0 mg/L), or the combination of both auxins. Shoots were grown under the same light and temperature conditions as shoot cultures obtained via clonal propagation. After eight weeks, the number and the length of induced roots were observed.

### 4.5. Callus Induction and Maintenance

Fragments of stems, leaves, and roots of micropropagated plantlets were used for callus initiation. Explants were transferred to 150-cm^3^ Erlenmeyer flasks containing 30 cm^3^ of the solidified basal medium consisting of MS nutrients with dicamba (Dic, 1.0 mg/L), or picloram (Pic, 0.5 mg/L; 1.0 mg/L; 2.0 mg/L), or 2,4-dichlorophenoxyacetic acid (2,4-D, 2.0 mg/L), or 2,4-D (2.0 mg/L) and Kin (0.2 mg/L; 0.5 mg/L), or 2,4-D (2.0 mg/L) and 1-naphthaleneacetic acid (NAA, 0.2 mg/L). Callus induced on explants was removed and transferred to a new medium of the same PGRs composition. Callus cultures were passaged every five weeks. In order to stabilize culture, fragments that were developing well were selected during the passage. After the morphological evaluation, the callus lines with the fastest growth were selected and the rate of biomass growth was calculated for the three following passages (VII, VIII, IX). The initial (FW0) and the final (FWX) weights of the callus lumps were measured. The growth index (GI) was calculated after three passages during three consecutive subcultures according to the following formula: GI = [(FWX − FW0)/FW0] × 100%. The flasks were stored in a darkroom. The following MS media were used in the experiment of callus stabilization: supplemented with Pic 1.0 mg/L or Pic 2.0 mg/L as well as with 2,4-D 2.0 mg/L and NAA 0.5 mg/L.

### 4.6. Flow Cytometry

Leaves of seedlings and in vitro shoots multiplied on MS medium, which was optimal for micropropagation, were used for the nuclear DNA content estimation. *Petunia hybrida* P × Pc6 (2.85 pg/2C) Marie and Brown, 1993) served as an internal standard. The samples were prepared as previously described [48], by simultaneous chopping of leaves of a sample and an internal standard in 1.0 mL of cold nuclei isolation buffer [49] supplemented with 1% (*v*/*v*) polyvinylpyrrolidone (PVP-10), propidium iodide (PI; 50 µg/cm^3^) and ribonuclease A (50 µg/cm^3^). For each sample, at least 5000 nuclei were analyzed directly after preparation, using the CyFlow Ploidy Analyzer flow cytometer (Sysmex Partec). Histograms were analyzed using the CyView 1.6 computer program. The analyses were replicated three times for each plant material. The coefficient of variation (CV) of G0/G1 peak of *L. borealis* ranged from 3.51 to 5.11%. The nuclear DNA content was calculated using the linear relationship between the ratio of the G0/G1 peak positions *L. borealis*/*Petunia* on a histogram of fluorescence intensities.

### 4.7. UPLC-HESI-HRMS Phytochemical Screening

Dry material, namely leafy shoots of intact plants, shoots and roots of micropropagated plantlets and callus biomass (50 mg), was extracted with 1.5 mL of cold 80% methanol for 12h at 4 °C, and then centrifuged (14,000 rpm, 4 °C, 10 min). The obtained supernatant was filtered through a PTFE membrane filter (14 mm, 0.22 µm, Kinesis) into glass HPLC vials (Agilent).

The Aquity UPLC (Waters, Milford, MA, USA) with the high resolution Orbitrap mass spectrometer (Thermo Fischer, Bremen, Germany) were used for the analysis of the methanolic extract of *Linnea borealis*. Two microliters of the sample were injected onto the BEH C13 column (1.7 µm of diameter, 2.1 × 150 mm) and separated with the use of 0.1% formic acid (LC-MS grade, Fluka) in ultrapure water (solvent A, MiliQ, Merck, Darmstadt, Germany) and acetonitrile (solvent B, LC-MS grade, Merck) at the flow rate of 0.300 µL/min and column temperature of 50 °C. The gradient program consisted of 2% B, 12 min—30% B, 15 min—98% B, 17 min—98% B, 18 min—2% B, and isocratic 2% B to 20 min. The PDA detector was operated at 250, 270, 330, and 360 nm of the wavelength. The heated electrospray ion source (HESI-II) settings were the following: capillary voltage −2.5 kV (negative), +3.5 kV (positive), sheath gas flow—35, auxiliary gas flow—10, sweep gas flow—3 arbitrary units, ion transfer tube temperature—400 °C, auxiliary gas heater temperature 350 °C, and S-lens RF level—50. The spectra were recorded at mass resolution of 70,000 FWHM in *m*/*z* range of 150–1500 and at 200 ms maximum injection time for full-MS scans and resolution of 17 500 FWHM, and at 50 ms maximum injection time for data dependent MS2 scans (top 5).

### 4.8. Statistical Analysis

The collected biotechnological data were subjected to a one-way analysis of variance (ANOVA) followed by Duncan’s POST-HOC test. ANOVA and the subsequent Student’s test were used for the flow cytometric results analysis. A two-sided *p*-value of 0.05 was applied to declare statistical significance. All the analyses were conducted employing STATISTICA v. 13 (StatSoft, Inc., Kraków, Poland, 2015).

## 5. Conclusions

The introduction of the rare and protected species *L. borealis* L. var. *borealis* into in vitro cultures enabled rapid clonal multiplication. This study demonstrated the influence of the type of the plant explant, hormonal supplementation in the medium and culture system on shoot multiplication and root development. The most efficient multiplied shoot biomass may be obtained from numerous lateral buds developed from multi-node stem segments in agitated liquid culture, in accordance with the biology of this species. A vigorously growing callus also ensures a good source of plant biomass and may be useful to obtain cell suspension culture in the future. In vitro technique can be used as a nondestructive approach for producing secondary metabolites from the homogenous biomass of medicinally important plants.

The preliminary phytochemical studies confirmed the presence of phenolic acids and flavonoid compounds in the species and have demonstrated the presence of iridoids and triterpenoid saponins for the first time. The chemical profile of European twinflower suggests the potential uses of both plant material from intact plants and biomass from in vitro cultures as a source of bioactive compounds with the confirmed pharmacological activity. Multiplied biomass with the profiled phytochemical composition of valuable secondary metabolites could be used for the biological studies of pharmacological interest.

Collection of in vitro plantlets may be also concerned as an ex situ conservation strategy for this rare European taxon.

## Figures and Tables

**Table 1:** The influence of the type of the plant explant and hormonal supplementation on *Linnaea borealis* var. *borealis* shoot multiplication on the solid MS medium.

MS Medium Supplementation			Explants (Mean No. of New Shoots ± SE)		
Cytokinin (mg/L)	Auxin (mg/L)	Gibberellin (mg/L)	Double Shoots	Single Shoot with Apical Meristem	Shoot Segments with One Node
-	-	-	2.46 ± 0.22 ^cd^	2.00 ± 0.17 ^c^	5.46 ± 0.31 ^ab^
BAP 1.0	IAA 0.1	-	4.15 ± 0.28 ^bc^	3.00 ± 0.19 ^b^	5.67 ± 0.36 ^ab^
BAP 0.5	IAA 0.1	GA_3_ 1.0	3.19 ± 0.50 ^cd^	2.75 ± 0.25 ^b^	5.25 ± 0.33 ^abc^
BAP 1.0	IAA 0.1	GA_3_ 1.0	4.95 ± 1.49 ^b^	2.58 ± 0.15 ^bc^	3.92 ± 0.36 ^d^
BAP 1.5	IAA 0.1	GA_3_ 1.0	5.12 ± 0.71 ^b^	3.25 ± 0.25 ^b^	6.13 ± 0.64 ^a^
BAP 2.0	IAA 0.1	GA_3_ 1.0	7.63 ± 0.73 ^a^	4.10 ± 0.32 ^a^	4.50 ± 0.61 ^bcd^
Kin 1.0	IAA 0.1	GA_3_ 1.0	2.16 ± 0.22 ^c^	2.58 ± 0.19 ^bc^	4.07 ± 0.40 ^cd^
Kin 2.0	IAA 0.1	GA_3_ 1.0	2.39 ± 0.20 ^c^	2.67 ± 0.19 ^bc^	6.33 ± 0.43 ^a^

BAP N^6^-benzylaminopurine; GA_3_ indole-3-acetic acid; IAA indole-3-acetic acid; MS Murashige and Skoog medium; Mean values with the same letter are not significantly different at *p* = 0.05 using Duncan’s Multiple Range test.

**Table 2:** The influence of the type of the plant explant (single, double, triple shoots) on *Linnaea borealis* var. *borealis* shoot multiplication on the solid MS medium with BAP 1.0 mg/L + IAA 0.1 mg/L + GA_3_ 1.0 mg/L.

Explants	Number of New Shoots per Explant ± SE	Number of New Shoots per One Shoot ± SE
1/single shoot	5.63 ± 0.63 ^b^	5.63 ± 0.63 ^a^
2/double shoot	10.89 ± 0.55 ^a^	5.37 ± 0.26 ^a^
3/triple shoot	10.79 ± 0.53 ^a^	3.51 ± 0.20 ^b^

BAP N^6^-benzylaminopurine; GA_3_ indole-3-acetic acid; IAA indole-3-acetic acid; MS Murashige and Skoog medium; Mean values with the same letter are not significantly different at *p* = 0.05 using Duncan’s Multiple Range test.

**Table 3:** The influence of the type of the plant explant on *Linnaea borealis* var. *borealis* shoot multiplication in the liquid MS medium with BAP 1.0 mg/L + IAA mg/L + GA_3_ 1.0 mg/L.

Explants	Number of New Shoots per Explant ± SE	Number of New Shoots per One Shoot ± SE	Shoots Length Increase (%) LI ± SE
1/single shoot	17.46 ± 0.91 ^b^	17.46 ± 0.91 ^b^	154.77 ± 15.16 ^a^
2/double shoot	48.80 ± 3.67 ^a^	24.11 ± 1.77 ^a^	150.36 ± 19.33 ^a^
3/triple shoot	50.30 ± 5.25 ^a^	15.72 ± 1.75 ^b^	138.14 ± 8.25 ^b^

BAP N^6^-benzylaminopurine; GA_3_ indole-3-acetic acid; IAA indole-3-acetic acid; MS Murashige and Skoog medium; LI length index. Mean values with the same letter are not significantly different at *p* = 0.05 using Duncan’s Multiple Range test.

**Table 4:** The comparative statement on *Linnaea borealis* var. *borealis* shoot multiplication in different systems with the application of MS medium with BAP 1.0 mg/L + IAA 0.1 mg/L + GA_3_ 1.0 mg/L.

In Vitro System	Explants	Number of New Shoots per Explant ± SE	Number of New Shoots per One Shoot ± SE
Solid medium	1/single shoot	5.63 ± 0.63 ^d^	5.63 ± 0.63 ^c^
Liquid medium	1/single shoot	17.46 ± 0.91 ^b^	17.46 ± 0.91 ^b^
Solid medium	2/double shoot	10.89 ± 0.55 ^c^	5.37 ± 0.26 ^c^
Liquid medium	2/double shoot	48.80 ± 3.67 ^a^	24.11 ± 1.77 ^a^
Solid medium	3/triple shoot	10.79 ± 0.53 ^c^	3.51 ± 0.20 ^d^
Liquid medium	3/triple shoot	50.30 ± 5.25 ^a^	15.72 ± 1.75 ^b^

BAP N6-benzylaminopurine; GA_3_ indole-3-acetic acid; IAA indole-3-acetic acid; MS Murashige and Skoog medium; Mean values with the same letter are not significantly different at *p* = 0.05 using Duncan’s Multiple Range test.

**Table 5:** The influence of auxins on *Linnaea borealis* var. *borealis* shoot rooting.

Medium	IAA (mg/L)	IBA (mg/L)	Induction (%)	Root Number ± SE	Root Length (cm) ± SE
¼ MS	-	-	50	4.57 ± 0.91 ^de^	2.01 ± 0.11 ^b^
½ MS	-	-	50	5.14 ± 1.03 ^de^	1.56 ± 0.08 ^cde^
MS	-	-	30.77	1.50 ± 0.50 ^e^	1.32 ± 0.26 ^efg^
MS	1.0	-	38.46	1.60 ± 0.40 ^e^	0.95 ± 0.19 ^gh^
MS	2.0	-	76.92	2.50 ± 0.50 ^e^	1.96 ± 0.13 ^bc^
MS	3.0	-	76.92	4.44 ± 0.85 ^de^	1.94 ± 0.14 ^bc^
MS	4.0	-	33.33	3.25 ± 0.63 ^e^	1.44 ± 0.21 ^def^
MS	2.0	5.0	94.74	17.22 ± 1.91 ^ab^	0.99 ± 0.04 ^gh^
MS	2.0	0.5	76.92	4.60 ± 0.91 ^de^	2.45 ± 0.13 ^a^
MS	1.0	2.0	100	19.78 ± 2.62 ^a^	0.67 ± 0.03 ^h^
MS	1.0	1.0	50	4.13 ± 0.83 ^de^	1.76 ± 0.10 ^bcd^
MS	1.0	0.5	50	2.00 ± 0.82 ^e^	1.68 ± 0.25 ^bcde^
MS	0.5	2.0	100	16.00 ± 2.97 ^ab^	0.91 ± 0.06 ^gh^
MS	-	1.0	100	10.00 ± 1.15 ^cd^	0.97 ± 0.05 ^fg^
MS	-	2.0	100	16.08 ± 2.25 ^ab^	1.17 ± 0.05 ^fg^
MS	-	3.0	100	13.36 ± 1.36 ^bc^	1.91 ± 0.07 ^bc^
MS	-	4.0	100	19.30 ± 1.86 ^a^	1.00 ± 0.04 ^gh^

IAA indole-3-acetic acid; IBA indole-3-butyric acid; MS Murashige and Skoog medium; Mean values with the same letter are not significantly different at *p* = 0.05 using Duncan’s Multiple Range test.

**Table 6:** The nuclear DNA content in leaves of *Linnaea borealis* var. *borealis* obtained from seedlings and micropropagated plantlets.

Plant Material	No. of Samples	DNA Content (pg/2C ± SE)
Seedling (Control)	3	1.778 ± 0.011 ^ns^
Plantlets (S1–S2)	12	1.783 ± 0.021

Control—leaves from seedling; S1, S2, S3, S4—leaves from shoots growing on the solid MS medium with BAP 1.0 mg/L, IAA 0.1 mg/L and GA_3_ 1.0 mg/L; ^ns^ Mean values are not significantly different at *p* = 0.05 using Student’s *t*-test.

**Table 7:** The influence of medium supplementation on growth of callus of *Linnaea borealis* var. *borealis* during the next three passages (VII–IX).

MS Medium Supplementation	Callus Growth Index (%) ± SE		
	Passage VII	Passage VIII	Passage IX
Pic 1.0 mg/L	379.67 ± 45.51 ^ab^	485.78 ± 21.77 ^a^	345.61 ± 21.61 ^b^
Pic 2.0 mg/L	408.99 ± 31.24 ^a^	468.66 ± 10.64 ^a^	525.80 ± 18.46 ^a^
2,4-D 2.0 mg/L + NAA 0.5 mg/L	281.40 ± 35.18 ^b^	311.56 ± 23.67 ^b^	380.83 ± 26.74 ^b^

2,4-D 2,4-dichlorophenoxyacetic acid; MS Murashige and Skoog medium; NAA 1-naphthaleneacetic acid; Pic (picloram) 4-Amino-3,5,6-trichloropicolinic acid; Mean values with the same letter are not significantly different at *p* = 0.05 using Duncan’s Multiple Range test.

**Table 8:** Secondary metabolites identified in plant biomass of *Linnaea borealis* var. *borealis* (leafy shoots from natural sites (NS); shoot cultures (SC); roots from micropropagated plantlets (R); biomass from callus cultures (C).

No	rt (min)		[M − H]^−^				[M + H]^+^				Name	Plant Material			
	UPLC-MS	UPLC-PDA	*m*/*z*	d (ppm)	Formula	Fragmentation	*m*/*z*	d (ppm)	Formula	Fragmentation					
												C	R	SC	NC
1	2.23	1.13	191.0550	−2.74	C_7_H_11_O_6_^−^	nd	nd			nd	Quinic acid (A)	+	+	+	+
2	4.53	3.43	218.1028	0.49	C_9_H_17_NO_5_^−^	146.0816, 88.0390, 71.0121	nd			nd	Pantothenic acid (A)	+	+	+	+
3	3.32	2.22	577.1349	0.50	C_30_H_25_O_12_^−^	407.0768, 289.0717, 245.0813, 125.0229	579.1490	2.18	C_30_H_27_O_12_	409.0899, 287.0527, 127.0391	Procyanidin (F)	nd	+	+	+
4	3.49	2.39	577.1348	1.50	C_30_H_25_O_12_^−^	407.0768, 289.0717, 245.0813, 125.0229	579.1493	1.56	C_30_H_27_O_12_	409.0899, 287.0527, 127.0391	Procyanidin (F)	nd	nd	+	+
5	4.23	3.13	289.0718	2.17	C_15_H_13_O_6_^−^	245.0816, 179.0340, 151.0388, 125.0228, 109.0279	291.0853	5.38	C_15_H_15_O_6_	207.0646, 139.0392, 123.0430	Catechin (F)	nd	nd	+	+
6	4.58	3.48	609.1462	0.50	C_27_H_29_O_16_^−^	447.0927, 285.0402	611.1589	3.84	C_27_H_31_O_16_	287.0554	Luteolin-3′,7-di-*O*-glucoside (F)	+	+	+	+
7	4.71	3.61	577.1344	0.60	C_30_H_25_O_12_^−^	407.0768, 289.0717, 245.0813, 125.0229	579.1494	1.56	C_30_H_27_O_12_	409.0899, 287.0527, 127.0391	Procyanidin (F)	nd	nd	+	+
8	5.76	4.66	609.1459	0.63	C_27_H_29_O_16_^−^	301.0353, 300.0275	611.1594	2.94	C_27_H_31_O_16_	303.0471	Quercetin-3-*O*-rutinoside (F)	nd	+	+	+
9	5.94	4.84	593.1516	1.53	C_27_H_29_O_15_^−^	285.0403, 269.0450	595.1666	−0.55	C_27_H_31_O_15_	nd	Luteolin-7-*O*-rhamnoglucoside I (F)	nd	+	+	+
10	6.02	4.92	463.0887	2.29	C_21_H_19_O_12_^−^	301.03519, 300.02737	465.1028	1.06	C_21_H_21_O_12_	303.0471	Quercetin-3-*O*-glucoside (F)	nd	+	+	+
11	6.23	5.13	593.1516	1.80	C_27_H_29_O_15_^−^	285.0403, 284.0323	595.1669	−1.06	C_27_H_31_O_15_	287.0554	Kaempferol-3-*O*-rutinoside (F)	nd	+	+	+
12	6.25	5.15	463.0887	2.29	C_21_H_19_O_12_^−^	301.03519, 300.02737	465.1028	1.06	C_21_H_21_O_12_	303.0471	Quercetin-3-*O*-galactoside (F)	nd	+	+	+
13	6.11	5.01	447.0928	1.30	C_21_H_19_O_11_^−^	285.0404, 284.0328	449.1079	1.20	C_21_H_21_O_11_	287.0554	Kaempferol-3-*O*-glucoside (F)	nd	nd	+	+
14	6.34	5.24	563.1409	1.51	C_26_H_27_O_14_^−^	269.0455, 133.0274	565.1566	−1.57	C_26_H_29_O_14_	433.1159, 271.0610	Apigenin-7-*O*- apioglucoside	+	nd	+	+
15	6.72	5.62	447.0928	1.30	C_21_H_19_O_11_^−^	285.0413, 133.0381	449.1079	1.20	C_21_H_21_O_11_	287.0554	Luteoloin-7-*O*-glucoside (F)	nd	+	+	+
16	6.71	5.61	593.1515	1.90	C_27_H_29_O_15_^−^	285.0403, 269.04623, 257.04605, 151.00226	595.1669	−1.01	C_27_H_31_O_15_	287.0554	Luteoloin-*O*- rhamnoglucoside II (F)	nd	+	+	+
17	6.50	5.40	577.1574	0.70	C_27_H_29_O_14_^−^	269.0454	579.1696	3.15	C_27_H_31_O_14_	271.0609	Apigenin-7-*O*- rhamnoglucoside (F)	nd	+	+	+
18	6.80	5.70	431.0981	1.74	C_21_H_19_O_10_^−^	269.0455	433.1129	1.26	C_21_H_21_O_10_	271.061	Apigenin-7-*O*-glucoside (F)	nd	+	+	+
19	7.98	6.88	447.0928	1.30	C_21_H_19_O_11_^−^	285.0403	449.1078	1.20	C_21_H_21_O_11_	287.0554	Luteolin-7-*O*-glucoside (F)	nd	nd	+	+
20	8.13	7.03	285.0404	3.77	C_15_H_9_O_6_^−^	241.0508, 217.0504, 175.0383, 151.0023, 133.0281	287.0542	4.65	C_15_H_11_O_6_	153.0199, 137.0958, 135.0938	Luteolin (F)	nd	+	+	+
21	9.10	8.00	269.0457	4.57	C_15_H_9_O_5_^−^	151.002	271.0592	5.35	C_15_H_11_O_5_	153.0175, 119.0488	Apigenin (F)	nd	+	+	+
22	9.26	8.16	285.0405	3.98	C_15_H_9_O_6_^−^	270.2250, 257.0460, 151.0026	287.0544	4.02	C_15_H_11_O_6_	nd	Kaempferol (F)	nd	nd	+	+
23	9.42	8.32	299.0558	0.86	C_16_H_11_O_6_^−^	284.0325	301.0699	4.37	C_16_H_13_O_6_	nd	Kaempferide (F)	nd	+	+	+
24	4.09	2.99	373.1138	2.27	C_16_H_21_O_10_^−^	211.0604, 193.0493, 167.0701, 149.0594, 123.0436	375.1276	4.07	C_16_H_23_O_10_	213.0742, 195.0644, 177.0546, 167.0704	Swertiamarin (I)	nd	nd	+	+
25	4.11	3.01	375.1294	0.72	C_16_H_23_O_10_^−^	213.0761, 169.0858, 151.0752, 125.0752, 119.0335, 113.0228	377.1439	2.21	C_16_H_25_O_10_	215.0914, 197.0428, 179.0694, 151.0756, 109.0650	Loganic acid (I)	nd	+	+	+
26	4.27	3.17	375.1294	0.72	C_16_H_23_O_10_^−^	213.0764, 195.0652, 151.0751	377.1433	3.83	C_16_H_25_O_10_	215.0914, 197.0428, 153.0537, 127.0391, 111.0806	Epi-loganic acid (I)	nd	+	+	+
27	4.21	3.11	435.1508	1.13	C_18_H_27_O_12_^−^ [M + FA − H]^−^	227.0918, 191.0550, 127.0385, 101.0227	391.1591	3.39	C_17_H_27_O_10_	229.1074, 211.0972, 179.0709, 151.0395, 109.0650	Loganin (I)	nd	+	+	+
28	4.62	3.52	373.1138	2.51	C_16_H_21_O_10_^−^	267.0658, 239.0716, 211.0753, 193.0495, 149.0594	375.1279	3.35	C_16_H_23_O_10_	213.0743, 195.0642, 177.0546, 167.0712, 151.0388, 149.0600, 133.0288, 125.0233, 107.0493	Geniposidic acid (I)	nd	+	+	+
29	5.14	4.04	403.1246	1.47	C_17_H_23_O_11_^−^	223.0595, 165.0545, 121.0279	427.1203	3.19	C_17_H_24_O_11_Na	265.0668, 255.0840, 233.0420, 195.0270	Secoxyloganin (I)	+	+	+	+
30	5.16	4.06	357.1192	1.80	C_16_H_21_O_9_^−^	195.06508, 125.02290	381.1151	2.77	C_16_H_22_O_9_Na	255.0829, 219.0623, 185.0404, 149.0203	Sweroside (I)	nd	+	+	+
31	5.47	4.37	403.1247	1.72	C_17_H_23_O_11_^−^	nd	405.1379	4.42	C_17_H_25_O_11_	211.0967, 193.0843, 177.0546, 161.0598, 151.0389	Gardenoside (I)	nd	+	+	+
32	5.81	4.71	387.1298	1.80	C_17_H_23_O_10_^−^	225.0759, 193.0496, 181.0494, 155.0336, 123.0435, 113.0228, 101.0228	389.1437	2.76	C_17_H_25_O_10_	209.0801, 177.0543, 165.0547, 151.0386, 107.0493	Secologanin (I)	nd	+	+	+
33	5.97	4.87	359.1349	1.98	C_16_H_23_O_9_^−^	nd	361.1484	4.15	C_16_H_25_O_9_	nd	Deoxyloganic acid (I)	nd	+	+	+
34	7.15	6.05	537.1614	1.16	C_25_H_29_O_13_^−^	375.1288, 179.0338, 161.0231, 135.0436	nd		nd	nd	Grandifloroside (I)	+	+	+	+
35	7.54	6.44	585.2195	1.96	C_27_H_37_O_14_^−^	373.1134, 211.09676, 193.0497, 149.05943	nd		nd	nd	Unknown iridoid I (I)	nd	nd	+	+
36	7.98	6.88	583.2036	1.59	C_27_H_35_O_14_^−^	373.1143, 209.0815, 193.0497, 149.0595	nd		nd	nd	Unknown iridoid II (I)	nd	+	+	+
37	3.43	2.33	299.0771	1.30	C_13_H_15_O_8_^−^	137.0229, 93.0329	301.0920	1.20	C_13_H_17_O_8_	nd	Hydroxybenzoic acid hexoside I (P)	+	+	+	+
38	3.58	2.48	359.0981	3.44	C_15_H_19_O_10_^−^	197.0446, 182.0211, 153.0544, 138.0308	nd			nd	Dimethoxy-hydroxybenzoic acid hexoside (P)	nd	+	+	+
39	3.67	2.57	315.0723	2.20	C_13_H_15_O_9_^−^	153.0544, 123.0436, 109.0279	nd			nd	Dihydroxybenzoic acid hexoside (P)	+	+	+	+
40	3.95	2.85	341.0875	2.80	C_15_H_17_O_9_^−^	179.0339, 135.0437	nd			nd	Caffeic acid hexoside (P)	nd	+	+	+
41	3.81	2.71	299.0771	1.30	C_13_H_15_O_8_^−^	137.0229	nd			nd	Hydroxybenzoic acid hexoside II (P)	+	+	+	+
42	3.92	2.82	353.0879	3.25	C_16_H_17_O_9_^−^	191.0551, 179.0339, 161.0233, 135.0437	355.1021	2.28	C_16_H_19_O_9_	163.0389, 145.0283, 135.0439, 117.0342	3-Caffeoylquinic acid (P)	+	+	+	+
43	3.34	2.24	339.0718	2.31	C_15_H_15_O_9_^−^	177.0182	341.0879	−1.91	C_15_H_17_O_9_	179.0331, 151.0758	Esculin (P)	nd	nd	+	+
44	4.36	3.26	353.0875	3.25	C_16_H_17_O_9_^−^	191.0551	355.1022	2.08	C_16_H_19_O_9_	163.0389, 145.0283, 135.0439	5-Caffeoylquinic acid (P)	+	+	+	+
45	4.88	3.78	353.0875	3.25	C_16_H_17_O_9_^−^	191.0551, 179.0339, 173.04428, 161.0233, 135.0437	355.1021	2.42	C_16_H_19_O_9_	163.0389, 145.0283, 135.0439	4-Caffeoylquinic acid (P)	+	+	+	+
46	4.93	3.83	353.0875	3.25	C_16_H_17_O_9_^−^	191.0551	355.1022	2.06	C_16_H_19_O_9_	163.0389, 145.0283, 135.0439	Cis-5-Caffeoylquinic acid (P)	nd	+	+	+
47	4.99	3.89	337.0929	3.26	C_16_H_17_O_8_^−^	191.0551	339.1069	3.23	C_16_H_19_O_8_	195.0641, 177.0547, 165.0539, 147.0437, 119.0491	3-Coumaroyl quinic acid (P)	nd	+	+	+
48	5.36	4.26	367.1038	2.32	C_17_H_19_O_9_^−^	193.0497, 191.0551, 173.0444	369.1175	2.90	C_17_H_21_O_9_	177.0546, 145.0283, 117.033	Feruloylquinic acid (P)	nd	+	+	+
49	5.49	4.39	337.0930	3.26	C_16_H_17_O_8_^−^	191.0551	339.1071	2.58	C_16_H19O_8_	147.0437, 119.0491, 91.0542	5-Coumaroyl quinic acid (P)	nd	+	+	+
50	5.77	4.67	367.1038	2.32	C_17_H_19_O_9_^−^	191.0551	369.1173	3.47	C_17_H_21_O_9_	177.0546, 135.0439	Feruloylquinic acid (P)	nd	+	+	+
51	6.33	5.23	515.1201	2.10	C_25_H_23_O_12_^−^	353.0883, 335.0776, 179.0338, 173.0446, 161.0229, 135.0436	517.1340	2.76	C_25_H_25_O_12_	163.0389, 145.0283, 135.0439	3,4-Caffeoylquinic acid (P)	nd	+	+	+
52	6.46	5.36	515.1191	1.55	C_25_H_23_O_12_^−^	353.0876, 353.0876, 191.0551, 179.0339, 173.0444	517.1335	2.18	C_25_H_25_O_12_	163.0389, 145.0283, 135.0439	3,4-Caffeoylquinic acid (P)	nd	+	+	+
53	6.58	5.48	515.1191	0.36	C_25_H_23_O_12_^−^	353.0877, 191.0551, 179.0339, 135.0437	517.1334	2.41	C_25_H_25_O_12_	163.0389, 145.0283, 135.0439	3,5-Caffeoylquinic acid (P)	nd	+	+	+
54	6.56	5.46	579.2079	1.89	C_28_H_35_O_13_^−^	417.1554, 402.1318, 387.1084, 181.0495, 166.0259	nd			nd	S(8-8)S hexoside (P)	nd	+	+	+
55	6.84	5.74	515.1191	0.37	C_25_H_23_O_12_^−^	353.0877, 191.0551, 179.0339, 135.0437	517.1335	2.18	C_25_H_25_O_12_	163.0389, 145.0283, 135.0439	3,5-Caffeoylquinic acid (P)	nd	+	+	+
56	7.57	6.47	515.1193	0.70	C_25_H_23_O_12_^−^	353.0879, 191.0551, 179.0339, 173.0444, 135.0437	517.1334	2.29	C_25_H_25_O_12_	163.0389, 145.0283, 135.0439	4,5-Caffeoylquinic acid (P)	nd	+	+	+
57	7.13	6.03	499.1249	1.81	C_25_H_23_O_11_^−^	337.0934, 173.0445, 163.0389	nd			nd	3-Caffeoyl-5-coumaroylquinic acid (P)	nd	+	+	+
58	7.31	6.21	499.1249	1.81	C_25_H_23_O_11_^−^	353.0879, 337.0933, 191.0551, 179.0339, 163.0388, 135.0437	nd			nd	3-Caffeoyl-4-coumaroylquinic acid (P)	nd	+	+	+
59	7.48	6.38	499.1249	1.81	C_25_H_23_O_11_^−^	353.0879, 337.0933, 191.0551, 179.0339, 173.0444, 163.0388	nd			nd	5-Caffeoyl-4-coumaroylquinic acid (P)	nd	+	+	+
60	7.42	6.32	529.1361	1.81	C_26_H_25_O_12_^−^	367.1029, 353.0869, 193.0498, 191.0551, 179.0339, 173.0446, 135.0438	nd			nd	3-Caffeoyl-5-feruloylquinic acid (P)	+	+	+	+
61	7.72	6.62	529.1361	1.81	C_26_H_25_O_12_^−^	367.1029, 353.0869, 193.0498, 191.0551, 179.0339, 173.0446, 135.0438	nd			nd	4-Caffeoyl-5-feruloylquinic acid (P)	+	+	+	+
62	8.18	7.08	499.1249	1.81	C_25_H_23_O_11_^−^	353.0874, 337.0928, 191.0552, 179.0339, 173.0444	nd			nd	4-Caffeoyl-5-coumaroylquinic acid (P)	nd	+	+	+
63	8.42	7.32	179.0338	−3.49	C_9_H_7_O_4_^−^	135.0437	181.0494	3.78	C_9_H_9_O_4_	163.0389, 145.0282, 138.0437	Caffeic acid (P)	nd	nd	+	+
64	9.05	7.95	515.1199	2.04	C_25_H_23_O_12_^−^	353.0879, 191.0551, 179.0339, 173.0444, 135.0437	517.13342	2.29	C_25_H_25_O_12_	nd	4,5-Caffeoylquinic acid (P)	nd	+	+	+
65	8.71	7.61	1235.6069	0.69	C_59_H_95_O_27_^_^	nd	nd			nd	Macranthoidin A (T)	nd	+	+	+
66	9.10	8.00	927.4966	1.40	C_47_H_75_O_18_^−^	603.3903, 453.3357	nd			nd	Akebiasaponin D (T)	nd	+	+	+
			973.5060	1.54	C_48_H_77_O_20_^−^ [M + FA]−	603.3888						nd	+	+	+
67	10.21	9.11	1073.5542	0.89	C_53_H_85_O_22_^−^	nd	nd			nd	Loniceroside C (T)	nd	+	+	+
68	11.32	10.22	957.5079	2.12	C_48_H_77_O_19_^−^	749.4479, 587.3954, 455.3544	nd			nd	Bourneioside B (T)	nd	+	+	+
69	11.83	10.73	1057.523	1.87	C_52_H_81_O_22_^−^	687.4111, 567.3679	nd			nd	Unknown Saponin I (T)	nd	+	+	+
70	12.54	11.44	911.4990	1.34	C_47_H_75_O_17_^−^	749.4475, 893.4863, 849.4981, 749.4475, 705.4574, 687.4468, 603.3901, 541.3889, 471.3448	nd			nd	Unknown Saponin II (T)	nd	+	+	+
71	12.60	11.50	765.4407	0.15	C_41_H_65_O_13_^−^	603.3917, 471.3464	nd			nd	Cauloside C (T)	+	+	+	+
72	12.60	11.50	811.4491	1.43	C_42_H_67_O_15_^−^	nd	nd			nd	Unknown Saponin III (T)	nd	+	+	+
73	13.21	12.11	749.4494	0.93	C_41_H_65_O_12_^−^	587.3946, 569.3834, 455.3531	nd			nd	Alpha-Hederin (T)	nd	+	+	+
74	13.85	12.75	603.3904	1.14	C_35_H_55_O_8_^−^	557.3862, 453.3366	nd			nd	Cauloside A (T)	nd	+	+	+
75	14.83	13.73	795.4532	1.43	C_42_H_67_O_14_^−^	nd	nd			nd	Unknown Saponin IV (T)	nd	+	+	+
76	15.14	14.04	471.3478	0.86	C_30_H_48_O_4_^−^	453.3356	nd			nd	Hederagenin (T)	+	+	+	+
77	15.66	14.56	469.3325	1.63	C_30_H_46_O_4_^−^	nd	nd			nd	Gypsogenin (T)	+	+	+	+
78	17.32	16.22	455.3531	1.25	C_30_H_47_O_3_^−^	nd	nd			nd	Oleanonic acid (T)	+	+	+	+

Abbreviation: rt—retention time, *m*/*z*—mass to charge ratio, d—error between measured mass and calculated, nd—not detected. Compounds identified in plant biomass of *Linnaea borealis* var. *borealis* belong to the classes: A—acids; F—flavonoids; I—iridoids/terpene-*O*-hexosies; P—phenols; T—triterpenes.

## Data Availability

The data presented in this study are available from the authors.

## Supplementary Materials

The following are available online. Figure S1: 2-D chromatogram of Linnaea borealis var. borealis extract of leafy shoots from natural sites. Figure S2: 2-D chromatogram of Linnaea borealis var. borealis extract of shoot cultures. Figure S3: 2-D chromatogram of Linnaea borealis var. borealis extract of roots from micropropagated plantlets. Figure S4: 2-D chromatogram of *Linnaea borealis* var. *borealis* extract of biomass from callus cultures.

## Sample Availability

Samples of *Linnaea borealis* biomass are available from the authors.
