# Supplementary material for: Linnaea borealis L. var. borealis—In Vitro Cultures and Phytochemical Screening as a Dual Strategy for Its Ex Situ Conservation and a Source of Bioactive Compounds of the Rare Species
Source: Molecules. 2021 Nov 11;26(22):6823. doi: 10.3390/molecules26226823 (PMC8620648; doi:10.3390/molecules26226823)
Supplement: Supplementary file 1 [file molecules-26-06823-s001.zip › molecules-1409553-supplementary.pdf]

Supplementary Materials

Linnaea borealis L. var. *borealis*—In Vitro Cultures and Phytochemical Screening as a Dual Strategy for Its Ex Situ Conservation and a Source of Bioactive Compounds of the Rare Species

Barbara Thiem [1], Dariusz Kruszka [2], Natalia Turowska [1], Elwira Sliwinska [3], Viktor Berge [4] and Małgorzata Kikowska [1,*]

[1] Department of Pharmaceutical Botany and Plant Biotechnology, Poznan University of Medical Sciences, 14 Św. Marii Magdaleny, 61-861 Poznań, Poland
[2] Institute of Plant Genetics, Polish Academy of Sciences, 34 Strzeszyńska St., 60-479 Poznań, Poland
[3] Laboratory of Molecular Biology and Cytometry, Department of Agricultural Biotechnology, Bydgoszcz University of Science and Technology, 7 Prof. Kaliskiego Ave., 85-789 Bydgoszcz, Poland
[4] Institute of Clinical Medicine, University of Oslo, Trondheimsveien 235, 0586 Oslo, Norway
* Correspondence: kikowska@ump.edu.pl; Tel: +48-61-6687851

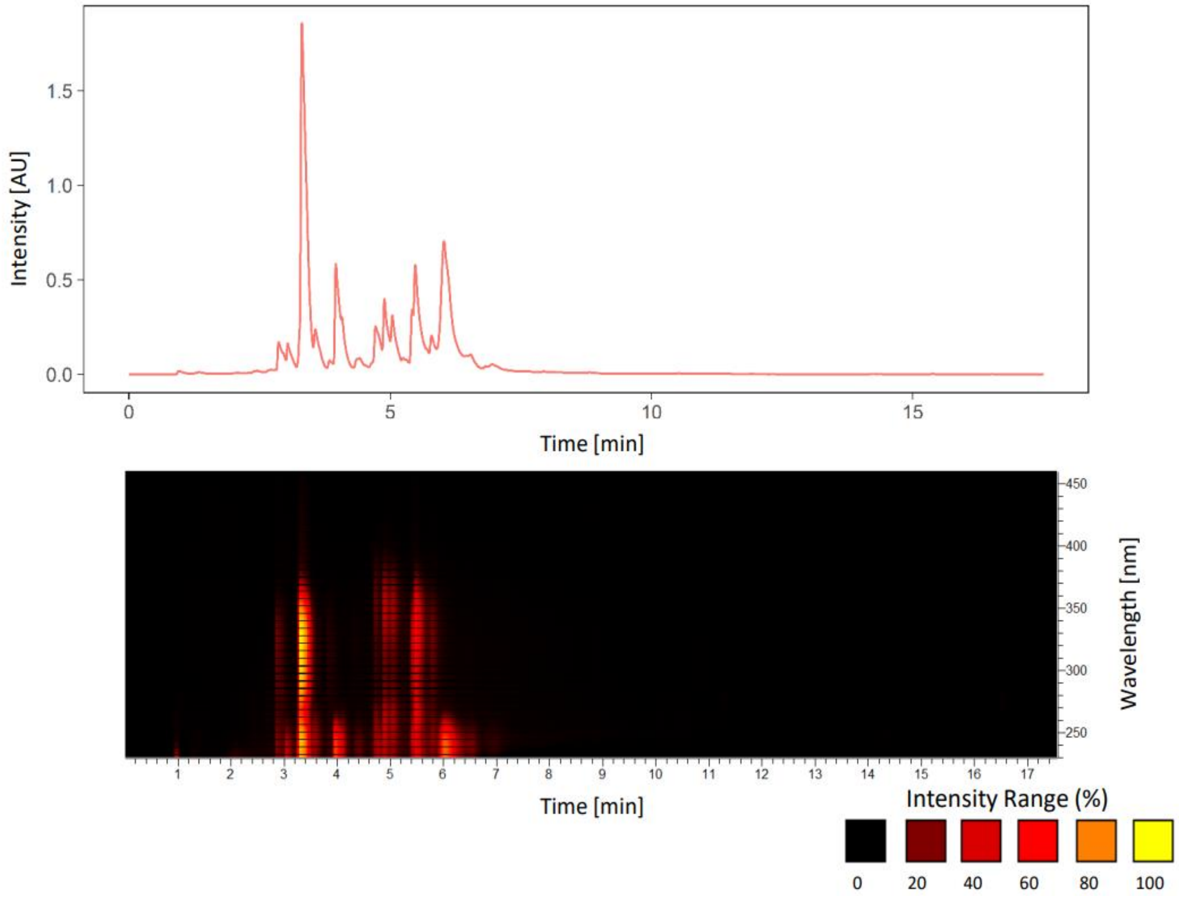

Figure S1. 2-D chromatogram of *Linnaea borealis* var. *borealis* extract of leafy shoots from natural sites.

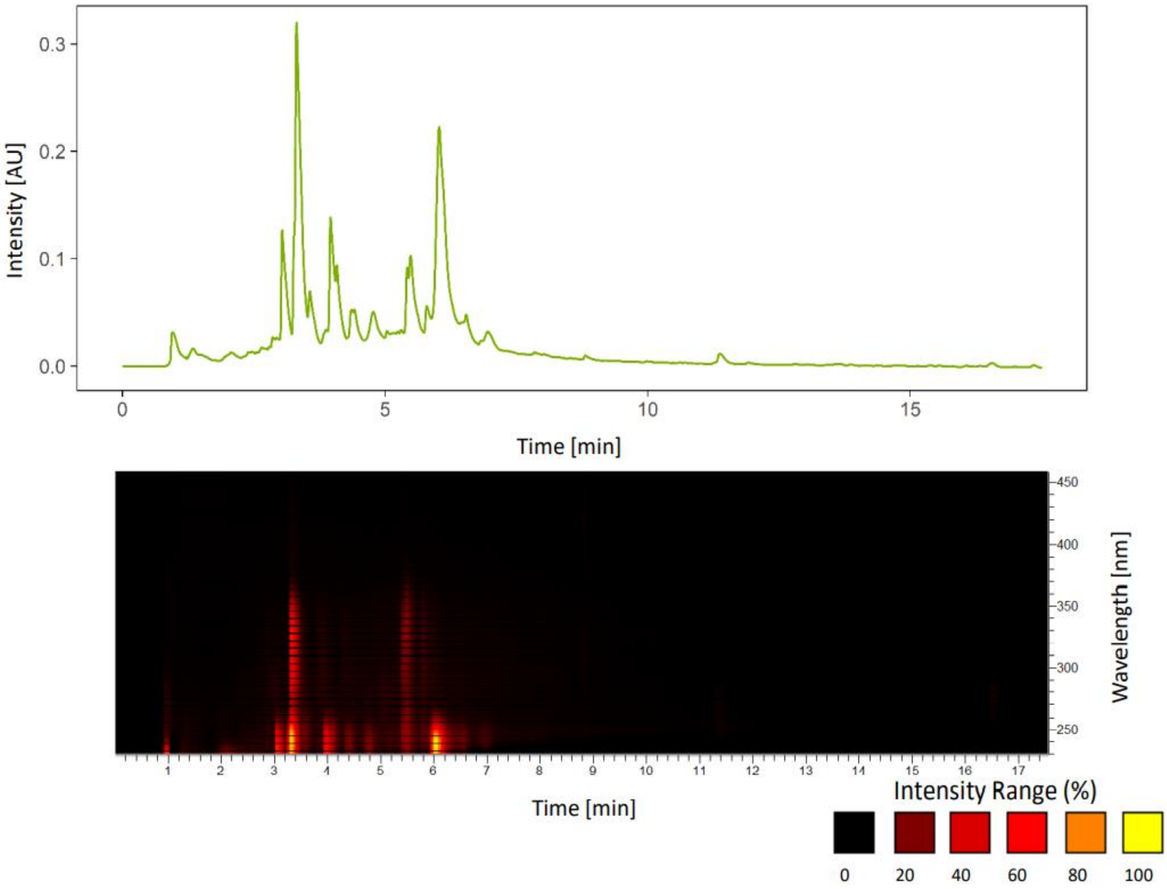

Figure S2. 2-D chromatogram of *Linnaea borealis* var. *borealis* extract of shoot cultures.

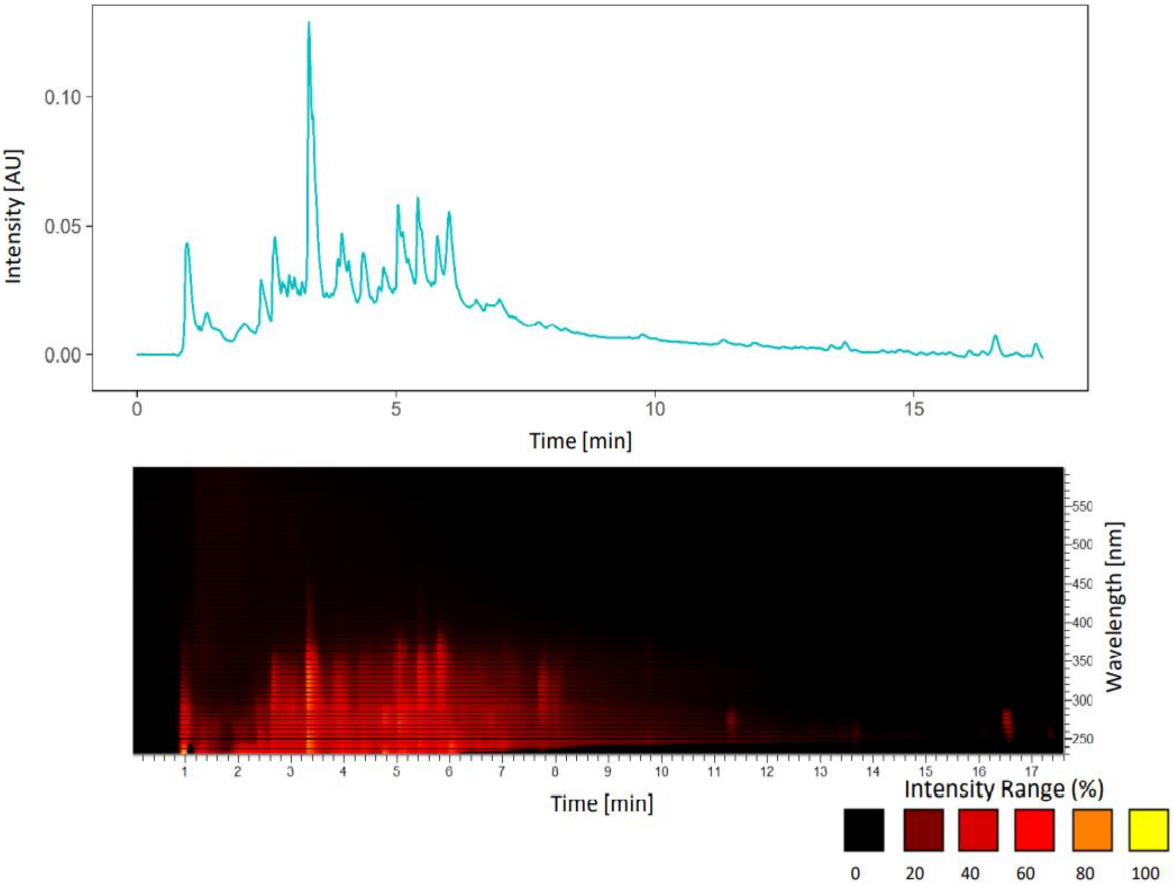

Figure S3. 2-D chromatogram of *Linnaea borealis* var. *borealis* extract of roots from micropropagated plantlets.

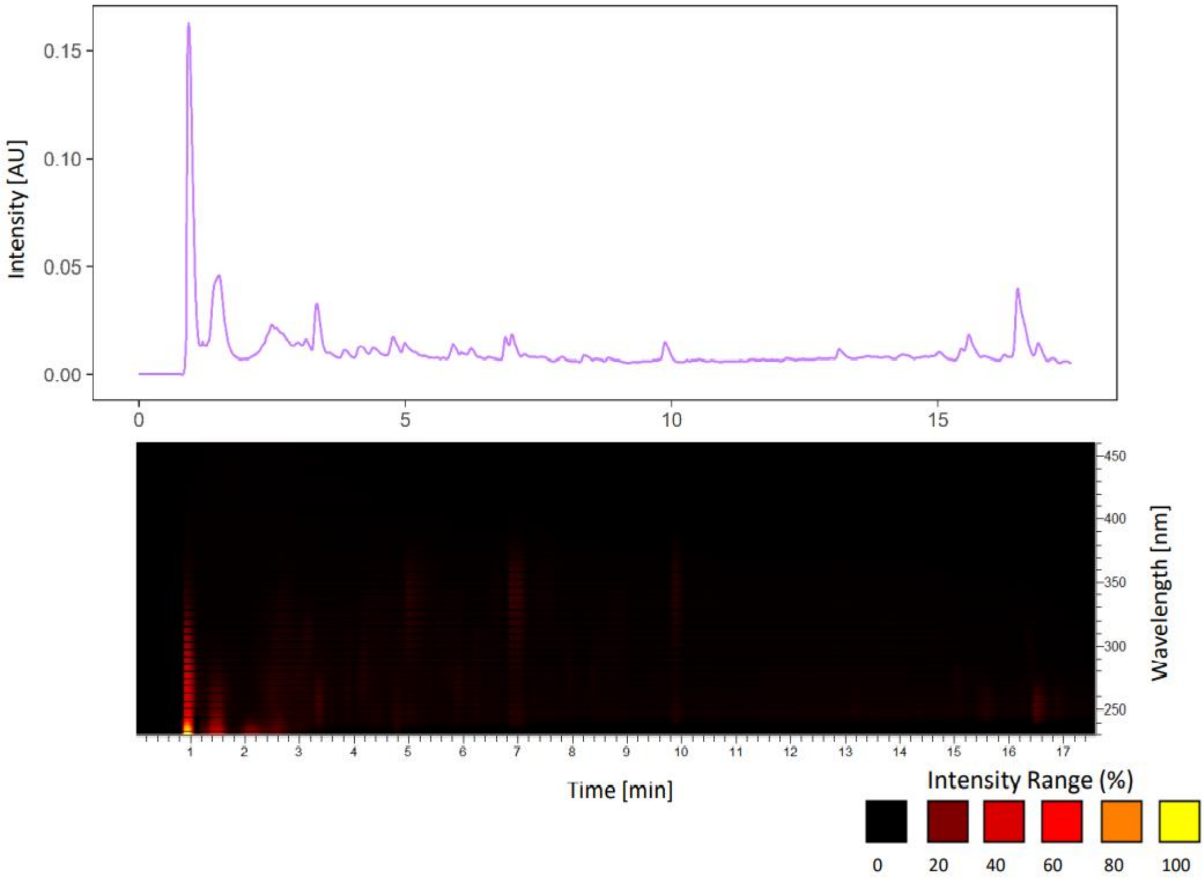

Figure S4. 2-D chromatogram of *Linnaea borealis* var. *borealis* extract of biomass from callus cultures.
